# Decreased Risk of Squamous Cell Carcinoma of the Head and Neck in Users of Nonsteroidal Anti-Inflammatory Drugs

**DOI:** 10.1155/2010/424161

**Published:** 2010-06-03

**Authors:** Neda Ahmadi, Radoslav Goldman, Françoise Seillier-Moiseiwitsch, Anne-Michelle Noone, Ourania Kosti, Bruce J. Davidson

**Affiliations:** ^1^Department of Otolaryngology Head and Neck Surgery, Georgetown University Hospital, Washington, DC 20007, USA; ^2^Lombardi Comprehensive Cancer Center, Department of Oncology, Georgetown University Medical Center, Washington, DC 20057, USA; ^3^Department of Biostatistics, Georgetown University Medical Center, Washington, DC 20057, USA

## Abstract

We evaluated the chemopreventive effect of nonsteroidal anti-inflammatory drug (NSAID) use in head and neck squamous cell carcinomas (HNSCC) by conducting a case-control study based on the administration of a standardized questionnaire to 71 incident HNSCC cases and same number of healthy controls. NSAID use was associated with a 75% reduction in risk of developing HNSCC. A significant risk reduction was noted in association with frequency of NSAID use. Restricting the analysis to aspirin users revealed a significant 90% reduction in risk of developing HNSCC. This study provides evidence for a significant reduction in the risk of developing HNSCC in users of NSAIDs, and specifically aspirin users.

## 1. Introduction

Head and neck cancer refers to carcinoma of the upper aerodigestive tract and the most common form is squamous cell carcinoma (HNSCC). In the United States alone, HNSCC accounts for 3% of newly diagnosed cancer cases [1–3] and the major known risk factors are smoking and chewing tobacco and alcohol consumption [4, 5], but recently the role of human papillomavirus (HPV) 16 has been highlighted [6]. Despite advances in treatment, patients with HNSCC develop recurrent disease, and 15% to 25% develop second primary malignancies within 5 years of initial diagnosis [7]. It is therefore crucial that chemopreventive measures other than tobacco and alcohol cessation should be further investigated.

One area in cancer chemoprevention which has gained significant recognition is the role of non-steroidal anti-inflammatory drugs (NSAIDs). The inhibitory effects of NSAIDs in HNSCC have been of interest since Panje [8] reported tumor regression of HNSCC in patients taking NSAIDs. The use of NSAIDs such as aspirin, indomethacin, and celecoxib has been associated with reduced risk of developing colorectal, esophageal, lung, gastric, breast, and ovarian cancers [9–15]. Randomized controlled trials and recent meta-analysis demonstrated that the use of aspirin decreases the incidence of colorectal adenomas and intraepithelial neoplasia in patients with previous history of colorectal cancer [15, 16]. Preliminary study supported the use of aspirin as adjuvant therapy to improve survival in subsets of postesophagectomy patients [17], while there was no evidence of a protective effect of aspirin and nonaspirin NSAID use in relation to esophageal adenocarcinoma in two independent studies [18, 19]. A protective effect of NSAID use and gastric noncardia adenocarcinoma but not with gastric cardia cancer has also been reported [18]. While the exact mechanism through which NSAIDs contribute to chemoprevention is incompletely understood, it is thought to involve the inhibition of the enzyme cyclooxygenase-2 (COX-2) of the arachadonic acid metabolism pathway [9]. There are only a few reported investigations of the chemopreventive effects of NSAIDs in HNSCC [11, 20–22] and the results have been inconsistent. The largest case-control study within the US examined 529 cancer patients and 529 hospital-based controls matched for age, gender, and smoking status. The authors demonstrated that the use of aspirin was associated with a 25% reduction in the risk of head and neck cancer [21]. 

In order to further evaluate the relationship between NSAIDs and chemoprevention in HNSCC, we performed a case-control study based on the administration of a standardized questionnaire examining NSAID use. The goal of our investigation was to determine whether the frequency of NSAID use is lower in patients presenting with established HNSCC compared to same age and gender controls.

## 2. Materials and Methods

### 2.1. Study Population and Data Collection

The study population consisted of HNSCC cases (*N* = 71) that were newly diagnosed and pathologically confirmed and recruited as part of an ongoing study at the Lombardi Comprehensive Cancer Center, at Georgetown University Medical School (GUMC) between 2003 and 2007. Inclusion criteria were age greater than 18, no previous history of malignancy, no prior treatment with chemoradiation or surgery, and fluency in English in order to consent to the study. Controls (*N* = 71) consisted of healthy individuals with no history of cancer that were either accompanying patients on their visit to Georgetown University Hospital or participated in the National Lung Screening Trial (NLST) at Georgetown University, a trial that aimed to evaluate radiological methods for detection of lung cancer in a population of cancer-free individuals. Participation rates were 65% for cases and 68% for controls. After informed consent was obtained, cases and controls received a structured, in-person interview assessing medical and cancer history, tobacco use, and alcohol use. Cases and controls were matched one to one on age within 10 years, gender, and history of tobacco use. A subject was considered an “ever smoker” if they had smoked >100 cigarettes in their lifetime and a “never smoker” if they responded negatively to the question. A subject was considered an “ever alcohol user” if they had consumed more than 12 alcoholic beverages per year in their entire life. Cumulative use of cigarettes and alcohol (pack-years, drink-years, resp.) was computed by multiplying the amount consumed per day by the number of years of usage. Information on occupational history, family medical history, recent nutritional supplements and caffeine intake, socioeconomic characteristics, and NSAID use history was also collected. More specifically, the questionnaire inquired about the frequency (never, occasional, daily) and reason of NSAID use (headache, arthritis, heart disease, stroke, or other). Among daily users, information on age at initiation of NSAID use, quantities, type, and change of patterns of use over time was collected, and this allowed calculation of a combined measure of dosage and duration by computing NSAID tablet years (tablets/day × years of use).

### 2.2. Data Analysis

Statistical analyses were carried out using the SAS software, version 9.2 (SAS Institute Inc. Cary, NC). For differences in demographic distributions and other characteristics between cases and controls, no statistical tests were performed because of the study design that involved 1 : 1 case: control pairing and would require matched pairs to be excluded if a variable is missing for any of the paired subjects. Characteristics of case and control groups separately stratified by NSAID use were compared using *t*-tests for continuous characteristics, Fisher's exact test for categorical characteristics with a cell count of 5 or less, and chi-square tests for all other categorical characteristics. The unadjusted and adjusted odds ratio (OR) and 95% Confidence Interval (CI) were estimated using conditional logistic regression. Marital status, educational level, and Body Mass Index (BMI) were considered to be potential confounders and added sequentially to the regression models.

## 3. Results

The characteristics of the participants are presented in Table 1. Cases and controls were matched on age, gender, and smoking status (Table 1). Ever alcohol consumption was comparable between cases and controls and so were pack- and drink-years, variables calculated to describe the cumulative use of cigarette smoking and alcohol use, respectively. Cases tended to have a lower BMI at the time of the interview compared to controls; however the BMI, as recalled by the participants 10 years later, was comparable between the two groups. Cases tended to have a lower educational level compared to controls and 76% of controls were married or living as married compared to 60% of cases. 

Subjects were categorized as either “never”, “occasional”, or “daily NSAID users”. The distribution of NSAID use varied between cases and controls, with more cases reporting never to have used NSAIDs and more controls reporting daily use of NSAIDs. Seventy percent of cases and 78% of controls who were daily users of NSAIDs were aspirin users (Table 1). The main reason for NSAID use in the control group was heart disease. The mean age at initiation of NSAID use was 56 for both cases and controls. Cases would more often have fewer than 2 NSAID tablet years (tablets/day × years of use) compared to controls.

Users and nonusers of NSAIDs were compared stratified by case status. These two groups did not differ in any of the parameters examined among controls but differed significantly with respect to age and marital status among cases (Table 2). More specifically, cases who were NSAID users tended to be older and were more likely to be married or be living as married. We also observed that cases who were NSAID users had a higher age-adjusted BMI compared to cases that were nonusers and would more likely be lighter drinkers as estimated by drink-years (Table 2).

The conditional logistic regression analysis showed that individuals that ever used NSAIDs had a 75% significant reduction in the risk of developing HNSCC (95%  CI = 33%–91%). Adjusting for potential confounders like educational level and marital status (Table 3) or BMI (results not shown) did not alter the risk estimates. A consistent risk reduction was noted in association with frequency of NSAID use when occasional and daily users were compared to never users (OR for occasional users = 0.37, 95%  CI = 0.13–1.04, OR for daily users = 0.12, 95%  CI = 0.04–0.42).

## 4. Discussion

Our data suggest that use of NSAIDs is associated with a significant 75% reduction in the risk of developing HNSCC in a frequency-dependent manner. As the majority of the study population was aspirin users, the data indicate that aspirin use, specifically, may be protective for HNSCC. Our findings corroborate those of a previous study by Jayaprakash et al. [21] and support the hypothesis that aspirin use is associated with a reduced risk of developing HNSCC. Despite the issues of temporality associated with the case-control design, our study provides evidence in favor of the potential protective effect of NSAIDs in general and aspirin more specifically. We cannot overlook the fact that the protective effect associated with NSAID use estimated in our analysis is somewhat higher than that already published previously [21] and that NSAID use may reflect the overall healthier lifestyle of the users. 

We examined the association of socioeconomic level, based on measures of income and education, and marital status with case status and NSAID use. In agreement with previous reports, we observed that participants that live with a partner were more likely to be cancer free and also be NSAID users compared to those that do not live with a partner. Studies have reported that married adults are healthier and live longer than unmarried adults [23, 24]. It is possible that among the more health conscious subjects that are also NSAID users, regular consumption of fruits and vegetables synergizes with NSAID use in lowering the risk of HNSCC. Cases were of lower educational level compared to controls. Given the documented associations between head and neck cancer and socioeconomic status, regular NSAID use could reflect a higher socioeconomic status, the latter acting as a confounder in the assessment of the effect of NSAID use on the risk of head and neck cancer [25–27]. Following adjustment for both marital status and educational level, the estimate of the protective effect of NSAIDs remained similar for daily users (OR = 0.12 and 95%  CI = (0.04, 0.42) before adjustment; OR = 0.14 and 95%  CI = (0.04, 0.54) after adjustment); it is therefore more likely that the observed association is real. Other surrogates of a healthy lifestyle like exercise and diet should be carefully examined in order to provide more precise estimates of the independent effect of NSAIDs in HNSCC risk. Moreover, the dose effects of NSAID use, smoking, and alcohol intake need to be considered in detail to further clarify the observed preventive effect. 

Leanness has been associated with an increased risk for cancers of the oral cavity and pharynx [28] and in our population cases had a lower BMI compared to controls. The observation was based on weight at the time of the interview; therefore, we cannot exclude the likelihood that the low BMI was the result of cancer development. Interestingly, cases that were NSAID users had an age-adjusted BMI higher than nonusers and comparable to the control group. The possibility that NSAID use in general, or aspirin in particular, among patients with head and neck cancer improves the quality of life of these patients needs to be investigated with great care.

As mentioned previously, it is believed that aspirin exerts a chemopreventive effect on other cancers through the inhibition of the enzyme COX-2. Cyclooxygenase-2 is a member of family of enzymes called prostaglandin endoperoxide (PGH) synthases that are involved with catalyzing the formation of prostaglandins (PGs) from the fatty acid arachadonic acid [29]. Both COX-2 activity and the products of arachadonic acid metabolism derived via this pathway have been correlated with the presence of HNSCC and tumor metastasis. Studies have demonstrated that the levels of COX-2 and its prostaglandin derivatives, specifically prostaglandin E_2_ (PGE_2_), are increased in premalignant and malignant lesions of HNSCC [30]. For instance, COX-2 messenger RNA is found to have increased activity in HNSCC mucosa versus normal mucosa, and PGE_2_ is found in increased levels in the tumor milieu and serum of patients with HNSCC [31, 32]. Elevated levels of PGE_2_ are also found in patients with higher stages of cancer and in those with tumor recurrence [21]. Therefore, there is overwhelming evidence supporting the increased activity of COX-2 in head and neck cancer patients. 

The overexpression of COX-2 and hence the increased levels of PGE_2_ in HNSCC patients could contribute to carcinogenesis via multiple mechanisms. First, through an increase in the levels of the antiapoptotic protein bcl-2, COX-2 overexpression promotes the survival of premalignant damaged cells leading to tumorigenesis [33]. Increased levels of COX-2 expression and PGE_2_ have also been shown to induce cell proliferation as well as angiogenesis by increasing the production of vascular-endothelial growth factor by epithelial cells [34]. Increased production of PGE_2_ also suppresses the immune system of patients with HNSCC by suppressing lymphokine production, T- and B-lymphocyte proliferation, and the cytotoxicity of natural killer cells and macrophages [33]. Metabolites such as PGE_2_, matrix metalloproteinase, and thromboxane are also thought to be associated with increased invasiveness and metastatic potential of squamous cell cancer [35, 36]. Therefore, through the inhibition of COX-2, NSAIDs reduce the production of PGE_2_ in HNSCC patients and block the downstream pathways that promote tumorigenesis. 

Animal model studies have also demonstrated the chemopreventive benefits of NSAIDs in HNSCC. A study by Tanaka et al. [37] involving rats with precancerous lesions of the tongue revealed that the incidence of HNSCC was reduced to 23%–31% with the administration of NSAIDs compared to 71% in rats that did not receive NSAIDs. Laboratory studies have also shown that NSAIDs increase the host immune cell infiltration in tumor cells [38], inhibit the growth of squamous cell cancer in mice [39–41], and cultured human HNSCC cell lines [42]. There has also been immunohistochemical evidence indicating that the expression of COX-2 protein in oral mucosal lesions increases as the lesions progress from hyperplasia to dysplasia with the highest levels of COX-2 stain found in SCC. These studies suggest that the inhibition of COX-2 by NSAIDs has a significant role in the chemoprevention of HNSCC. 

In 2002, the US Preventive Services Task Force strongly recommended that clinicians discuss aspirin use with adults who are at increased risk of coronary heart disease [43]. The recommendation was based on data from 5 randomized controlled trials that showed a 28% reduction in risk of myocardial infarctions with aspirin use. There is a large body of evidence supporting the chemopreventive role of NSAIDs in different types of cancer although studies that shed doubt on the chemopreventive effect of NSAIDs should not be overlooked. Very few case-control studies have investigated such an effect in HNSCC. Our study adds to this literature by demonstrating a statistically significant 75% reduction in the risk of developing HNSCC in patients using NSAIDs. We recommend caution when interpreting our data due to the possibility of an overestimated protective effect of NSAID use; we cannot exclude the possibility that people that use NSAIDs regularly have an overall healthier lifestyle compared to people that do not and that contributes to the protective effect observed. This study explored the chemopreventive effects of aspirin as the majority of the population examined was aspirin users. Despite its limitations and the case-control design, our research supports the hypothesis that the risk of HNSCC is reduced in users of NSAIDs especially daily users and underlies the need for further examination of the chemopreventive effect of NSAIDs in this disease.

## Figures and Tables

**Table 1:** Characteristics of patients with head and neck cancer and matched controls.

	Cases		Controls	
	(*N* = 71)		(*N* = 71)	
*Age*, mean (SD)	56	(12)	57	(9.7)
*Gender*, *N* (%)				
Male	61	(86)	61	(86)
Female	10	(14)	10	(14)
*Smoking Status*, *N* (%)				
Ever	55	(73)	55	(73)
Never	16	(27)	16	(27)
*Pack-years*, *N* (%)				
<40	14	(26)	15	(27)
41–60	16	(29)	11	(20)
>60	21	(38)	24	(44)
Missing	4	(7)	5	(9)
*BMI*, mean (SD)				
Current	26	(5.3)	28	(4.6)
10 years ago	24	(4.0)	24	(4.0)
*Alcohol Consumption*, *N* (%)				
Ever	71	(100)	65	(92)
Never	0	(0)	5	(7)
Missing	0	(0)	1	(1)
*Drink-years*, *N* (%)				
<20	18	(25)	16	(24)
21–40	17	(24)	13	(20)
41–60	9	(13)	9	(14)
>60	24	(34)	18	(28)
Missing	3	(4)	9	(14)
*Education level*, *N* (%)				
<College	38	(54)	17	(24)
≥College	33	(46)	53	(75)
Missing	0	(0)	1	(1)
*Married or living as married*, *N* (%)				
Yes	42	(60)	54	(76)
No	28	(39)	17	(24)
Missing	1	(1)	0	(0)
*NSAID use*, *N* (%)				
Never	25	(35)	10	(14)
Occasional	27	(38)	24	(34)
Daily	19	(27)	37	(52)
Type of NSAID *, *N* (%)				
Aspirin	14	(75)	29	(78)
Other	3	(16)	5	(14)
Missing	2	(11)	3	(8)
Reason for using NSAID *, *N* (%)				
Headache	2	(11)	0	(0)
Heart disease	9	(47)	24	(65)
Other	7	(37)	11	(30)
Missing	1	(5)	2	(5)
*Age of NSAID use initiation*, mean (SD)*	56	(9.4)	56	(9.3)
NSAID tablet years *, *N* (%)				
<2	6	(32)	7	(19)
2-3	0	(0)	7	(19)
>3	8	(42)	13	(35)
Missing	5	(26)	10	(27)

*Among daily users.

Missing data: Current BMI unknown for 2 subjects, 10 years ago unknown for 8 subjects, age at NSAID initiation unknown for 5 subjects.

SD: Standard Deviation.

**Table 2:** Characteristics of cases and controls by NSAID use. Number (percentage) is shown unless otherwise indicated.

	Cases					Controls				
	Never users (*N* = 25)		Ever users (N=46)		*P*-value*	Never users (*N* = 10)		Ever users (*N* = 61)		*P*-value*

*Age, years *mean (SD)	52	(12)	58	(11)	.02	54	(10)	58	(10)	.30
*Smoking Status*					.77					.68
Ever	20	(80)	35	(76)		7	(70)	48	(79)	
Never	5	(20)	11	(24)		3	(30)	13	(21)	
*Pack-years*					.54					.13
<40	6	(30)	8	(23)		3	(43)	12	(25)	
40–60	4	(20)	12	(34)		3	(43)	8	(17)	
≥60	9	(45)	12	(34)		1	(14)	23	(48)	
Unknown	1	(5)	3	(9)		0	(0)	5	(10)	
*Race*					.40					.11
Caucasian	19	(76)	40	(87)		8	(80)	47	(77)	
African American	5	(20)	4	(9)		0	(0)	10	(16)	
Other	1	(4)	2	(4)		2	(20)	4	(7)	
*Alcohol Consumption*					—					.55
Ever	25	(100)	46	(100)		9	(90)	56	(91)	
Never	0	(0)	0	(0)		1	(10)	4	(7)	
Unknown	0	(0)	0	(0)		0	(0)	1	(2)	
*Drink-years*					.06					.53
<20	4	(16)	14	(31)		3	(34)	13	(24)	
20–40	10	(40)	7	(15)		3	(33)	10	(18)	
40–60	1	(4)	8	(17)		1	(11)	8	(14)	
≥60	9	(36)	15	(33)		1	(11)	17	(30)	
Unknown	1	(4)	2	(4)		1	(11)	8	(14)	
*Current BMI, *mean (SE)^†^	24	(1.1)	27	(0.8)	.08	28	(1.5)	28	(0.6)	.97
*Married or living as married*					.01					0.99
Yes	10	(40)	32	(70)		8	(80)	46	(75)	
No	15	(60)	13	(28)		2	(20)	15	(25)	
Unknown	0	(0)	1	(2)		0	(0)	0	(0)	
*Education level*					.42					.99
<College	15	(60)	23	(50)		2	(20)	15	(24)	
≥College	10	(40)	23	(50)		8	(80)	45	(74)	
Unknown	0	(0)	0	(0)		0	(0)	1	(2)	

*P-values were computed using *t*-tests for continuous characteristics, Fisher's exact test for categorical characteristics with cell frequencies of 5 or less, and chi-square tests for all other characteristics. Missing values were excluded from the *P*-value computation.

^†^BMI comparisons were age adjusted. Means are shown for a 56-year-old subject.

Missing data: BMI unknown for 1 case and 1 control.

SD: Standard Deviation; SE: Standard Error.

**Table 3:** Unadjusted and Adjusted risk estimated of having Head and Neck Cancer by NSAID use categories.

	Cases/Controls	OR1	95% CI	*P*-value*	Cases/Controls	OR2	95% CI	*P*-value*
*NSAID use*								
Never	25/10	1.00			25/10	1.00		
Ever	46/61	0.25	(0.09, 0.67)	.01	44/59	0.31	(0.11, 0.88)	.03
Never	25/10	1.00			25/10	1.00		
Occasional	27/24	0.37	(0.13, 1.04)	.06	26/23	0.45	(0.15, 1.34)	.15
Daily	19/37	0.12	(0.04, 0.42)	.001	18/36	0.14	(0.04, 0.54)	.004
*Aspirin Use*								
Never User	15/6	1.00			15/6	1.00		
Daily Aspirin Use	10/19	0.10	(0.01, 0.78)	.03	10/19	0.15	(0.02, 1.3)	.09

*P-values were computed using conditional logistic regression.

OR1: Unadjusted Odds Ratio; OR2: Odds Ratio after adjusting for educational level and marital status.
